# Interaction of the *Streptomyces* Wbl protein WhiD with the principal sigma factor σ^HrdB^ depends on the WhiD [4Fe-4S] cluster

**DOI:** 10.1074/jbc.RA120.012708

**Published:** 2020-04-17

**Authors:** Melissa Y. Y. Stewart, Matthew J. Bush, Jason C. Crack, Mark J. Buttner, Nick E. Le Brun

**Affiliations:** ‡ Centre for Molecular and Structural Biochemistry, School of Chemistry, University of East Anglia, Norwich Research Park, Norwich, NR4 7TJ, United Kingdom; § Department of Molecular Microbiology, John Innes Centre, Norwich Research Park, Norwich, NR4 7UH, United Kingdom

**Keywords:** iron-sulfur protein, nitric oxide, protein-protein interaction, metalloprotein, microbiology, mass spectrometry (MS), sporulation, iron-sulfur cluster, sigma factor, Streptomyces, Wbl proteins, WhiD, bacterial gene regulation, protein dimerization

## Abstract

The bacterial protein WhiD belongs to the Wbl family of iron-sulfur [Fe-S] proteins present only in the actinomycetes. In *Streptomyces coelicolor*, it is required for the late stages of sporulation, but precisely how it functions is unknown. Here, we report results from *in vitro* and *in vivo* experiments with WhiD from *Streptomyces venezuelae* (*Sv*WhiD), which differs from *S. coelicolor* WhiD (*Sc*WhiD) only at the C terminus. We observed that, like *Sc*WhiD and other Wbl proteins, *Sv*WhiD binds a [4Fe-4S] cluster that is moderately sensitive to O_2_ and highly sensitive to nitric oxide (NO). However, although all previous studies have reported that Wbl proteins are monomers, we found that *Sv*WhiD exists in a monomer-dimer equilibrium associated with its unusual C-terminal extension. Several Wbl proteins of *Mycobacterium tuberculosis* are known to interact with its principal sigma factor SigA. Using bacterial two-hybrid, gel filtration, and MS analyses, we demonstrate that *Sv*WhiD interacts with domain 4 of the principal sigma factor of *Streptomyces*, σ^HrdB^ (σ^HrdB^_4_). Using MS, we determined the dissociation constant (*K_d_*) for the *Sv*WhiD-σ^HrdB^_4_ complex as ∼0.7 μm, consistent with a relatively tight binding interaction. We found that complex formation was cluster dependent and that a reaction with NO, which was complete at 8–10 NO molecules per cluster, resulted in dissociation into the separate proteins. The *Sv*WhiD [4Fe-4S] cluster was significantly less sensitive to reaction with O_2_ and NO when *Sv*WhiD was bound to σ^HrdB^_4_, consistent with protection of the cluster in the complex.

## Introduction

The WhiB-like (Wbl) family of [4Fe-4S] cluster-containing proteins is found exclusively in Actinobacteria, including soil dwelling *Streptomyces* bacteria, which are the most abundant source of clinically important antibiotics, and *Mycobacterium tuberculosis*, one of the world's most devastating pathogens. Actinobacteria typically contain multiple Wbl paralogues with distinct functions (1). In *Streptomyces*, Wbl proteins play key roles in sporulation (WhiB and WhiD) (2–5) and the regulation of antibiotic production (WblA) and resistance (WblC) (6–8). In Mycobacteria and other pathogens, Wbl proteins have been shown to play key roles in virulence and antibiotic resistance (7, 9, 10).

Wbl proteins, which can be divided into five distinct classes on the basis of sequence (1), are generally small (∼80–140 residues) and soluble and contain conserved Cx_n_Cx_2_Cx_5_C and G[V/I]WGG motifs (11). The Cys residues of the former act as ligands to a [4Fe-4S] cluster, whereas the latter motif has been proposed to be important for protein-protein interactions. The NMR structure of *M. tuberculosis* [4Fe-4S] WhiB1 was recently reported, the first for any Wbl protein (12), revealing a four-helix bundle with the [4Fe-4S] cluster coordinated at the interface of helices 1, 2, and 3 by the four conserved Cys residues. In the same study, it was also shown that WhiB1 forms a stable complex with the C-terminal part (domain 4) of SigA, the cell's major sigma factor (12), and other *M. tuberculosis* Wbl proteins have also been shown to interact with SigA (13, 14). Very recently, a high-resolution crystal structure of WhiB1 in complex with domain 4 of SigA (σ^A^_4_), was reported, revealing conformational changes of WhiB1 upon complex formation, particularly involving helix 4, which swings away from helix 2 by 120° toward helix 3 of WhiB1 in the complex structure. Unusually for interactions with sigma factors, the WhiB1-σ^A^_4_ interaction is dominated by hydrophobic interactions around the [4Fe-4S] cluster-binding pocket (15), accounting for why the cluster is essential for stability of the complex (12, 15).

The interaction of Wbl proteins with other proteins is also known; work in both *Streptomyces* and *Corynebacterium* revealed that WhiB controls the process of sporulation via a direct interaction with a (non-Wbl) transcription factor called WhiA (5, 16). Thus, evidence is accumulating that Wbl proteins function together with partner proteins.

Wbl [4Fe-4S] clusters are generally reactive toward O_2_/ROS and particularly the cytotoxin nitric oxide (NO), leading to suggestions that Wbl proteins might function as sensors of oxidative and/or nitrosative stress (2, 17–22). In *M. tuberculosis*, it has been shown that WhiB3 (which, like *Streptomyces* WhiD, is a class III Wbl) regulates the accumulation of triacylglycerol in response to hypoxia and NO exposure in activated macrophages (17, 18). Importantly, the interaction between WhiB1 and σ^A^_4_ was found to be unaffected by the presence of O_2_, but highly sensitive to NO (12). Reaction with NO led to dissociation of the complex and a form of WhiB1 that can bind its own promoter (12, 23).

Here, we report studies, using *in vivo* and *in vitro* approaches, of *S. venezuelae* WhiD (*Sv*WhiD), showing that, like its Mycobacterial homologues, it forms a complex with domain 4 of the principal sigma factor σ^HrdB^, which depends on the [4Fe-4S] cluster. Reaction of [4Fe-4S] WhiD with NO results in nitrosylation of the cluster and dissociation of the complex.

## Results and discussion

### Characterization of SvWhiD

**Figure 1.**
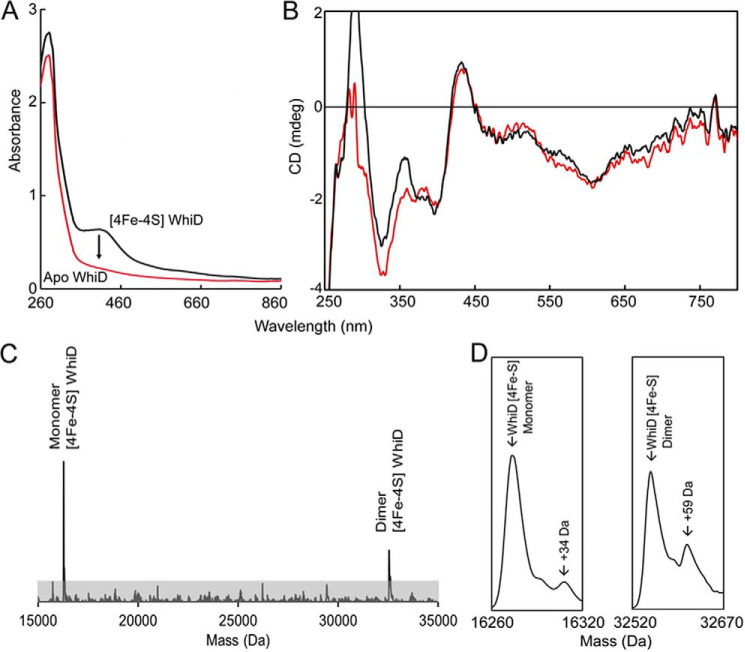
***S. venezuelae* WhiD (*Sv*WhiD) binds a [4Fe-4S] cluster.** *A* and *B*, UV-visible (*A*) and CD spectroscopic characterization (*B*) of *Sv*WhiD. *A*, the *black line* spectrum corresponds to that of as isolated *Sv*WhiD, whereas exposure of purified *Sv*WhiD to O_2_ results in the *red line* spectrum, recorded after centrifugation of the apoprotein sample, which was prone to precipitation. *B*, the CD spectrum of as isolated *Sv*WhiD is shown as a *red line*. Also plotted is the spectrum of a 2:1 mixture of σ^HrdB^_4_-WhiD, show in *black* demonstrating that the *Sv*WhiD cluster environment is not significantly affected by binding to σ^HrdB^_4_. The additional intensity below 350 nm is because of the aromatic residues of σ^HrdB^_4_. *C*, deconvoluted ESI-MS spectrum of *Sv*WhiD under nondenaturing conditions, containing peaks because of monomeric and dimeric forms. *D*, the monomeric and dimeric [4Fe-4S] *Sv*WhiD peaks plotted on an expanded mass scale. For absorbance and CD spectroscopy experiments, *Sv*WhiD was in 50 mm Tris, 300 mm NaCl, pH 7.2; for ESI-MS experiments, *Sv*WhiD (10 μm) was in 250 mm ammonium acetate, pH 7.2.

Anerobically purified His-tagged *Sv*WhiD was straw yellow in appearance with an absorbance spectrum typical of a [4Fe-4S] cluster protein, with a maximum located around 410 nm (Fig. 1*A*). Cluster loading was typically in the range ∼90%. CD spectroscopy detects cluster optical transitions and is particularly sensitive to the cluster environment. The spectrum in Fig. 1*B* is very similar to that previously reported for *S. coelicolor* WhiD (2), consistent with the presence of a [4Fe-4S] cluster in a similar environment. The LC-MS spectrum of purified *Sv*WhiD contained a major peak at 15,923 Da (predicted mass 15,924 Da) (Fig. S1), corresponding to the cluster-free (apo) protein with an N-terminal methionine cleavage. Two lower intensity peaks were also observed at 13,390 and 12,805 Da, corresponding to truncated forms of the protein, resulting from cleavage of 26 and 33 C-terminal residues, respectively, from the full-length protein. From LC-MS and SDS-PAGE (Fig. S1), the truncated forms were estimated to account for ∼10% of the total protein.

Nondenaturing ESI-MS, in which noncovalently bound cofactors are retained upon ionization, has been shown to be extremely useful for determining the nature of the cluster in [Fe-S] proteins (22, 24–27). Application here gave a deconvoluted spectrum containing a small peak at 15,919 Da corresponding to the apoprotein with all four cysteines in disulfide bridges. The major peak at 16,273 Da corresponded to [4Fe-4S] WhiD (predicted mass 16,273 Da) (Fig. 1*C* and 1*D*). The spectrum also contained a peak at 13,154 Da, corresponding to the [4Fe-4S]-bound form of one of the truncated forms of *Sv*WhiD (12,805 Da) (see Fig. S1), indicating that the 33 C-terminal residues are not important for cluster binding/stability.

Purification of WhiD under aerobic conditions also resulted in weakly colored fractions, consistent with previous findings that the *S. coelicolor* protein cluster is moderately resistant to O_2_-mediated degradation. The sample lost color entirely 30 min post purification, resulting in apoprotein, as indicated by the complete disappearance of the 410-nm absorbance band (Fig. 1*A*).

### SvWhiD exists in both monomeric and dimeric forms

**Figure 2.**
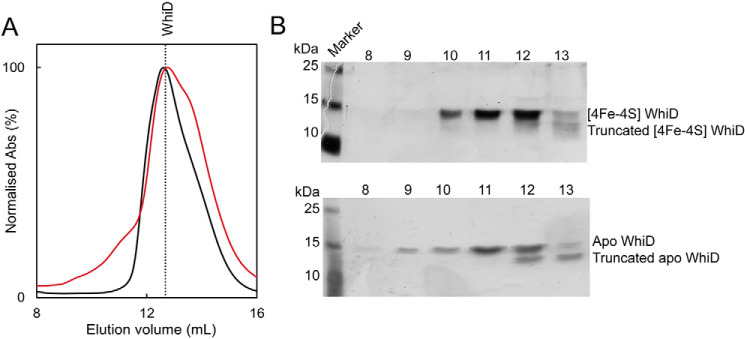
***Sv*WhiD exists in a monomer/dimer equilibrium.** *A*, gel filtration elution profiles (A_280 nm_) of holo-*Sv*WhiD (*black line*) and apo*Sv*WhiD (*red line*). *B*, SDS-PAGE analysis of elution fractions for holo-*Sv*WhiD, and apo*Sv*WhiD, spanning the elution volume 8–13 ml. *Sv*WhiD (45 μm) was in 50 mm Tris, 300 mm NaCl, pH 8.

*Sv*WhiD was found to elute from an analytical gel filtration column (Fig. 2*A*) as a broad peak with an elution volume indicative of a mass of ∼27 kDa, midway between the masses of monomeric and dimeric forms. A low-intensity shoulder on the low mass side of the peak was observed, representing the truncated forms of *Sv*WhiD. SDS-PAGE showed the full-length protein was present across the full elution profile, whereas the truncated forms were observed only in the lower mass fractions, indicating that they exist only as a monomer (Fig. 2*B*). ApoWhiD was also found to behave as a monomer-dimer mixture, but with some further higher mass species at lower elution volumes (Fig. 2, *A* and *B*). Nondenaturing ESI-MS revealed the presence of both monomer and dimer forms of [4Fe-4S] *Sv*WhiD (Fig. 1*C* and *D*). Together, the data indicate that the presence or absence of the cluster does not affect the association state of *Sv*WhiD. The absence of the cluster promotes the formation of higher mass forms of WhiD, which may arise from oxidation of Cys residues, resulting in crosslinking of *Sv*WhiD monomers.

### The C-terminal extension of SvWhiD is crucial for protein dimerization

The role of the C-terminal extension in *Sv*WhiD dimerization was explored further by generating a truncated form of WhiD which lacked 33 residues at the C terminus. Truncated *Sv*WhiD was found to elute from an analytical gel filtration column at a volume indicative of a monomer (Fig. S2), and SDS-PAGE confirmed the presence of truncated *Sv*WhiD across the elution profile. UV-visible absorbance and CD spectra revealed no significant differences between truncated and full-length *Sv*WhiD (Fig. S3), indicating that the absence of the C-terminal extension does not affect the cluster environment.

Nondenaturing ESI-MS revealed the presence of only monomeric truncated [4Fe-4S] WhiD (dimeric truncated [4Fe-4S] *Sv*WhiD was not observed using parameters optimized to observe dimeric full-length *Sv*WhiD (Fig. S3). Apo-truncated *Sv*WhiD was not observed because of high cluster load (>90%). Thus, we conclude that the C-terminal part of *Sv*WhiD is essential for dimerization.

A general conclusion from previous characterizations of Wbl proteins is that they are monomeric proteins (2, 12, 28), and so these findings for *Sv*WhiD were unexpected. We note that *Sv*WhiD has a C-terminal extension of 18 residues compared with *Sc*WhiD, and, although the proteins exhibit overall 78% sequence identity, only 1 of the last 33 residues of *Sv*WhiD is conserved in *Sc*WhiD (the proteins are 100% identical from residues 1–95) (Fig. S4). Therefore, the difference in the C-terminal part is likely to be functionally important. BLAST analysis revealed comparable *putative* WhiD sequences (sharing ≥60% identity with the *Sv*WhiD C-terminal extension) to be present in some *Streptomyces* species. Secondary structure analysis of *Sv*WhiD, using Phyre2 (29) suggested that the core of *Sv*WhiD resembles that recently reported for WhiB1 (12, 15), whereas the C-terminal extension is predicted to constitute an additional helix (*Sv*WhiD residues 104–121) (see Fig. S4).

### SvWhiD [4Fe-4S] cluster reacts with NO

**Figure 3.**
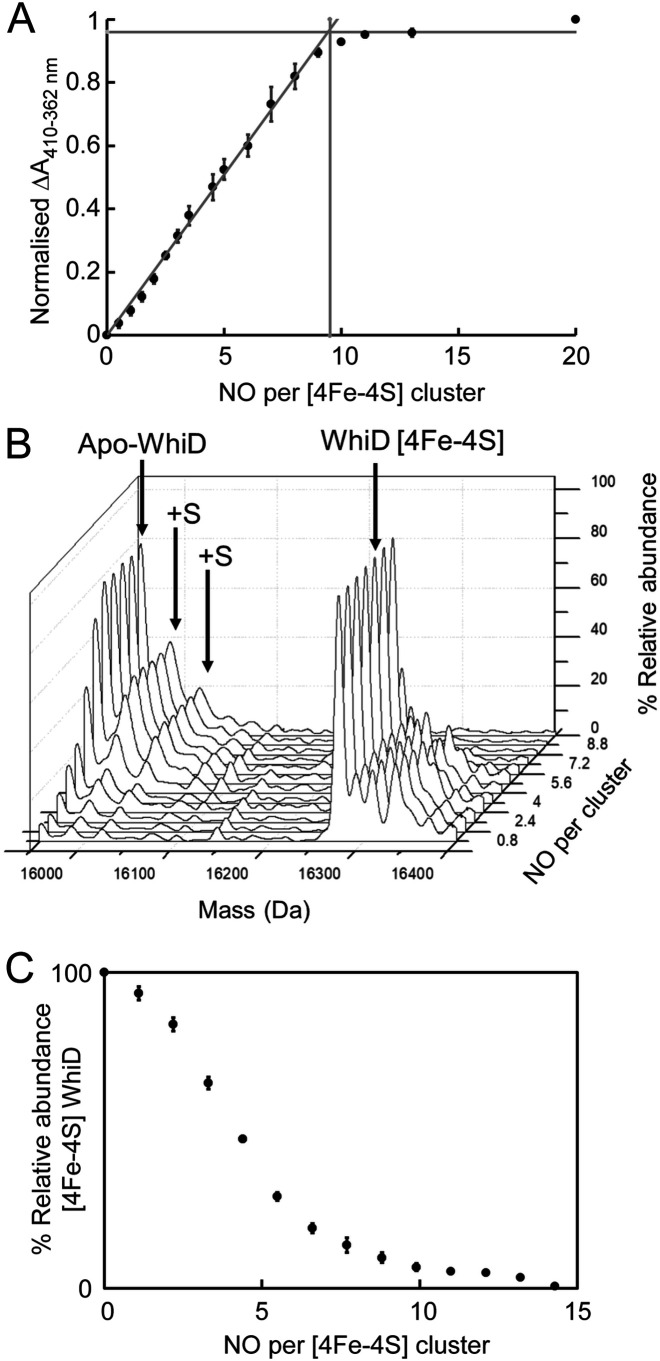
**Reaction of *Sv*WhiD with NO.** *A*, plot of normalized ΔA (410–362 nm) as a function of NO per cluster; data are shown in Fig. S2. Phases are indicated by *intersecting lines*. The *vertical line* indicates the number of NO molecules required for full reaction. *B*, titration of holo-*Sv*WhiD with NO (up to 14 NO per cluster) followed by nondenaturing ESI-MS. The 3D plot shows how the 2D spectrum changes with increasing NO. *C*, plot of percentage relative abundance of [4Fe-4S] *Sv*WhiD as a function of NO per cluster. *Error bars* represent S.E. from *n* = 2 experiments. For absorbance experiments, *Sv*WhiD (21 μm) was in 50 mm Tris, 300 mm NaCl, pH 7.2; for ESI-MS experiments, *Sv*WhiD (16 μm) was in 250 mm ammonium acetate, pH 7.2. Note that under the conditions of the ESI-MS experiments, no precipitation was observed upon reaction with NO.

Wbl proteins from a variety of species have been shown to react slowly with O_2_ but rapidly with ∼8–10 NO in a concerted manner, resulting in protein-bound iron-nitrosyl species (19–23). Therefore, the reaction of *Sv*WhiD with NO was investigated. Stepwise titration of [4Fe-4S] WhiD with PROLI-NONOate, to give 0–20 NO per cluster, resulted in mild precipitation, suggesting that the nitrosylation intermediates/products of *Sv*WhiD may be less stable than those of *Sc*WhiD (22). Scattering because of precipitation distorted the spectral changes upon nitrosylation (Fig. S5), but a shoulder at 362 nm was observed that is indicative of the formation of iron-nitrosyl species. Spectral changes could be more readily visualized by plotting the difference between absorbance at 410 and 362 nm as a function of NO per cluster (Fig. 3*A*). This shows that the reaction was complete at ∼9 NO per cluster, demonstrating the reaction of multiple NO molecules with each cluster, consistent with data previously reported for *S. coelicolor* WhiD (19).

The reaction was also investigated by nondenaturing ESI-MS. Deconvoluted mass spectra measured at increasing concentrations of NO (Fig. 3*B*) revealed increasing abundance of peaks because of apoWhiD, and one and two sulfur adducts of apoWhiD (+32 and +64 Da), along with the eventual loss of [4Fe-4S] WhiD. Absolute ion counts for these species were then used to determine percentage abundance of the [4Fe-4S] form as a function of NO per cluster (Fig. 3*C*). The plot is similar to that of absorbance data in Fig. 3*A* in that it indicates that ∼9–10 NO molecules are required for full reaction of the *Sv*WhiD [4Fe-4S] cluster. However, the plot is nonlinear, indicating that the reaction is not fully concerted such that the reaction of one cluster with NO does not go entirely to completion before the reaction at another cluster begins.

Interestingly, no nitrosylated forms of the WhiD [4Fe-4S] cluster, nor any iron-nitrosyl products, were detected by nondenaturing MS. Such species were recently detected by MS for *Sc*WhiD (22), suggesting that NO complexes of *Sv*WhiD are less stable under the conditions of the MS experiments than those of *Sc*WhiD.

### SvWhiD interacts specifically with the essential principal sigma factor σ^HrdB^

**Figure 4.**
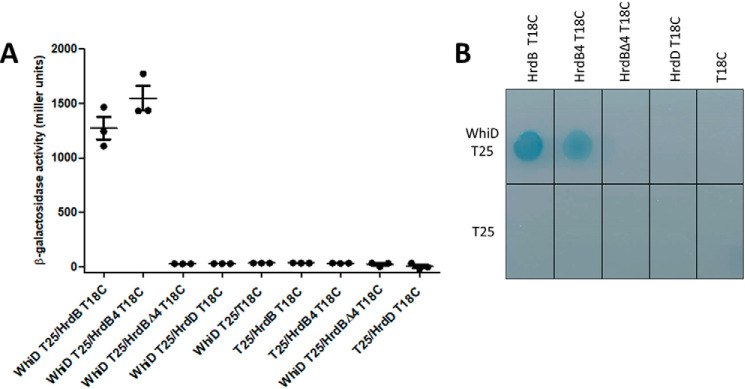
**Bacterial two-hybrid analysis of the interaction of *Sv*WhiD with the sigma factors full-length σ^HrdB^, domain 4 of σ^HrdB^ (*HrdB4*), σ^HrdB^ lacking domain 4 (*HrdB*Δ*4*) and full-length σ^HrdD^.** *A*, the listed pairs of constructs were transferred into the BACTH reporter strain *E. coli* BTH101 by transformation. Three independent clones were picked and subjected to β-gal assays. *Error bars* show the S.E. of the three replicates carried out for each pairwise combination. *B*, the corresponding strains spotted onto M63 minimal medium supplemented with lactose and X-gal. Interaction is indicated by growth and a *blue* color.

To gain insight into *Sv*WhiD function, we screened a bacterial adenylate cyclase two-hybrid (BACTH) (30) shotgun *S. venezuelae* sonicated DNA genomic library using *whiD* as bait, to look for interacting proteins. Five of the 13 positive clones isolated carried in-frame fusions to the same gene, *hrdB*, encoding the essential principal sigma factor, σ^HrdB^ (31–33). Furthermore, the five positive clones carried only the 3′ of the *hrdB* gene (Fig. S6). Among these five clones, the most C-terminal fusion started at Asp-488, corresponding to the beginning of domain 4 of σ^HrdB^ (Fig. S6), which is responsible for binding to the −35 element of target promoters. This suggested that interaction with WhiD was principally mediated by this domain. To confirm and extend this analysis, we used the BACTH system to measure the interaction of WhiD with (i) full-length σ^HrdB^, (ii) domain 4 alone, and (iii) σ^HrdB^ lacking domain 4. This analysis showed strong interaction of WhiD with full-length σ^HrdB^ and with domain 4 alone, but none with σ^HrdB^ lacking domain 4 (Fig. 4). These results confirmed that the interaction with *Sv*WhiD is principally mediated by domain 4 of σ^HrdB^. To determine whether this interaction is specific to the principal sigma factor, we also tested the interaction between WhiD and the closely related sigma factor σ^HrdD^ (32, 33). WhiD and σ^HrdD^ did not interact, suggesting that WhiD interaction is indeed specific for σ^HrdB^ (Fig. 4).

**Figure 5.**
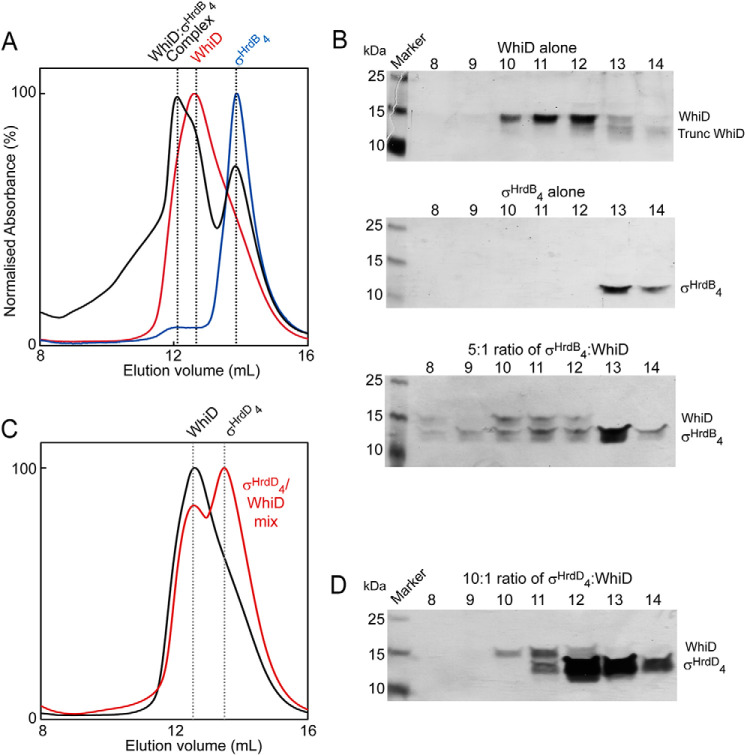
***Sv*WhiD binds to σ^HrdB^_4_ of *S. venezuelae* but not to domain 4 of an alternative sigma factor (σ^HrdD^_4_).** *A*, gel filtration elution profiles (A_280 nm_) of WhiD (*red line*), σ^HrdB^_4_ (*blue line*), and a mixture of WhiD and σ^HrdB^_4_ in a 1:5 ratio (*black line*). *B*, fractions were resolved by SDS-PAGE and visualized by silver staining. *Sv*WhiD (*top*), σ^HrdB^_4_ (*middle*), and the WhiD/σ^HrdB^_4_ mixture (*bottom*), spanning the elution volume 8–14 ml. *C* and *D*, gel filtration (*C*) and SDS-PAGE (*D*) analysis (*lower panel*) of the interaction of WhiD with σ^HrdD^_4_. *C*, the gel filtration elution profile (A_280 nm_) of a mixture of *Sv*WhiD and σ^HrdD^_4_ in a 1:10 ratio (*red line*) is plotted along that of *Sv*WhiD alone for comparison (*black line*). *D*, fractions spanning the elution volume 8–14 ml were analyzed by SDS-PAGE and silver stained. *Sv*WhiD (46 μm), σ^HrdB^_4_ (250 μm), and σ^HrdD^_4_ (500 μm) were in 50 mm Tris, 300 mm NaCl, pH 8. Note that the chromatogram for WhiD alone in (*A*) and (*C*) and most of the SDS-PAGE gel image for WhiD alone (*upper gel*) in (*B*) are also part of Fig. 2; they are included here to permit easy visual comparisons between WhiD alone and mixtures of WhiD and σ^HrdB^_4_ or σ^HrdD^_4_.

To complement the *in vivo* interaction data, domain 4 of the *S. venezuelae* sigma factor σ^HrdB^ (σ^HrdB^_4_), harboring the HTH-motif that binds to the −35 promoter element, was expressed and purified, resulting in a His-tagged protein of ∼11 kDa (Fig. S7). The lack of Trp and Tyr residues in this σ^HrdB^ domain resulted in a low extinction coefficient at 280 nm (ϵ = 2850 m^−1^ cm^−1^), and so a 5-fold excess of σ^HrdB^_4_ was mixed with *Sv*WhiD to enable detection of the protein through its absorbance upon elution from an anaerobic gel filtration column. The elution profile of the *Sv*WhiD/σ^HrdB^_4_ mixture contained peaks corresponding to the individual proteins, as judged from elution profiles of the individual proteins run down the column separately (Fig. 5*A*). However, also present was a peak at higher mass that was not observed in the elution profiles of the separate proteins. This peak corresponded to a mass of ∼35 kDa, too low to indicate a (WhiD)_2_-σ^HrdB^_4_ complex, but higher than that predicted for a monomeric WhiD-σ^HrdB^_4_ complex. Thus, it is apparent that the *Sv*WhiD monomer-dimer equilibrium described above remains a feature upon complex formation with σ^HrdB^_4_. Consistent with this, SDS-PAGE analysis of the gel filtration elution fractions demonstrated the presence of σ^HrdB^_4_ (and WhiD) across the high-mass fractions (Fig. 5*B*); equivalent fractions were devoid of σ^HrdB^_4_ for the separately run protein. SDS-PAGE also suggested the presence of a component of aggregated WhiD/σ^HrdB^_4_ at >50 kDa (Fig. 5*B*).

The specificity of complex formation with σ^HrdB^_4_ was tested by equivalent experiments with a protein containing σ^HrdD^_4_. *Sv*WhiD and σ^HrdD^_4_ were mixed in a 1:10 ratio (extinction coefficient for σ^HrdD^_4_ is ϵ_280 nm_ = 1490 m^−1^ cm^−1^, and so a higher concentration than that employed for σ^HrdB^_4_ experiments was used) and analyzed by gel filtration and SDS-PAGE. No evidence of an interaction between the proteins was observed; the elution profile of the WhiD/σ^HrdD^_4_ mixture was a superposition of the profiles of the individual proteins at ∼27 kDa and ∼11 kDa, with no higher mass complex (Fig. 5, *C* and *D*). Thus, *Sv*WhiD does not interact with domain 4 of σ^HrdD^.

**Figure 6.**
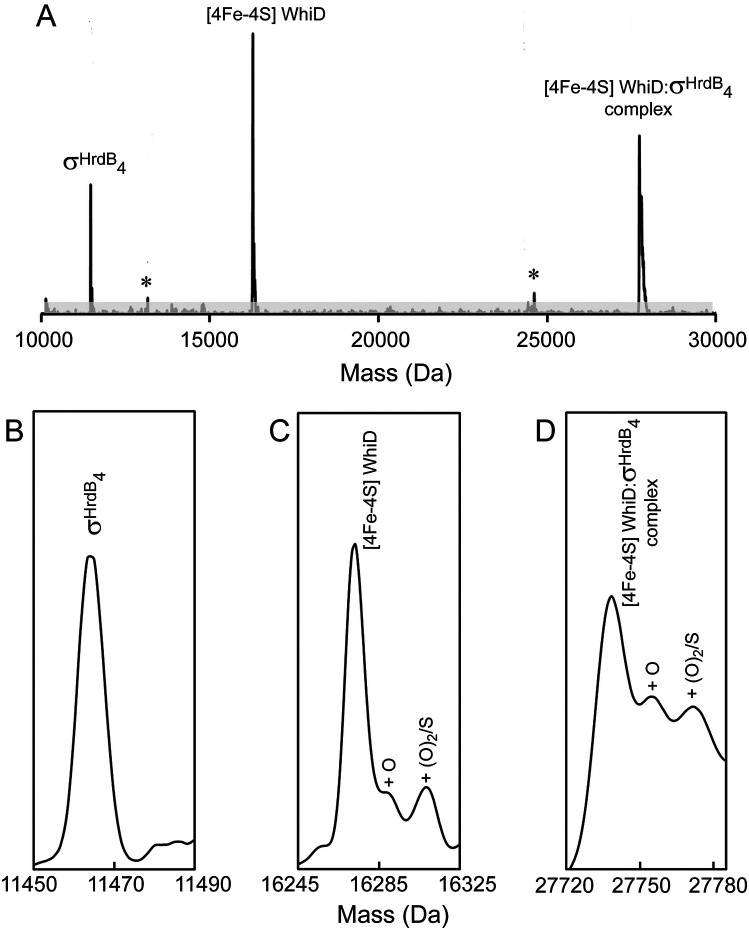
**ESI-MS analysis demonstrates binding of cluster-bound *Sv*WhiD to σ^HrdB^_4_.** *A*, deconvoluted mass spectrum measured under nondenaturing conditions of a solution containing a 2:1 ratio of σ^HrdB^_4_ to *Sv*WhiD containing σ^HrdB^_4_, [4Fe-4S] WhiD, and a σ^HrdB^_4_-[4Fe-4S] WhiD complex. *Asterisks* indicate truncated forms of [4Fe-4S] WhiD alone and in complex with σ^HrdB^_4_. *B–D*, show spectra of the three main species (σ^HrdB^_4_ (*B*), [4Fe-4S] WhiD (*C*), σ^HrdB^_4_-[4Fe-4S] WhiD complex (*D*)) plotted on an expanded mass scale, with oxygen/sulfur adducts indicated. *Sv*WhiD (5 μm) and σ^HrdB^_4_ (100 μm) were in 250 mm ammonium acetate, pH 7.2.

Nondenaturing MS was used to investigate the interaction between σ^HrdB^_4_ and *Sv*WhiD. The deconvoluted spectra of a 2:1 mixture of σ^HrdB^_4_ and *Sv*WhiD revealed the individual proteins, as well as a major peak corresponding to the mass of a WhiD [4Fe-4S]-σ^HrdB^_4_ complex, along with peaks because of oxygen and/or sulfur adducts (Fig. 6). Two minor species were also observed, at 13,154 Da and 24,619 Da, corresponding to the [4Fe-4S]-bound form of 33-residue truncated *Sv*WhiD alone and in complex with σ^HrdB^_4_. This indicates that the C-terminal part of WhiD is not important for interaction with the sigma factor. CD spectroscopy of a 2:1 WhiD/σ^HrdB^_4_ mixture showed that complex formation has no significant effect on the cluster environment (Fig. 1*B*).

**Figure 7.**
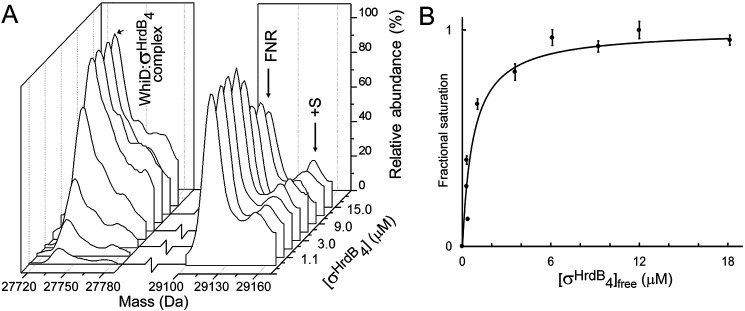
**Determination of *K_d_* for *Sv*WhiD-σ^HrdB^_4_ complex.** *A*, deconvoluted mass spectra under nondenaturing conditions of a solution containing *Sv*WhiD and increasing concentration of σ^HrdB^_4_, as indicated. An internal protein standard (FNR) was also present in the solution to enable quantification of the complex. *B*, plot of fractional saturation of *Sv*WhiD-σ^HrdB^_4_ complex formation as a function of the concentration of free σ^HrdB^_4_. Fractional saturation was determined from absolute ion counts because of the complex with reference to FNR ion counts. *Error bars* represent S.E. Data were fitted using a simple binding equation from which the dissociation constant, *K_d_*, was obtained directly. *Sv*WhiD (3 μm) and σ^HrdB^_4_ (0.75–21 μm) were in 250 mm ammonium acetate, pH 7.2.

The dissociation constant for the interaction between *Sv*WhiD and σ^HrdB^_4_ was determined using ESI-MS. Multiple samples were prepared containing an increasing concentration of σ^HrdB^_4_. To assist with quantification, each sample contained a fixed concentration of a protein standard, I151A FNR,^2^ which has a mass of 29,123 Da, close to that of the *Sv*WhiD-σ^HrdB^_4_ complex. *m/z* spectra (Fig. S8) were deconvoluted and the mass regions of the complex and protein standard plotted (Fig. 7*A*). Absolute ion counts for the complex and FNR standard were used to determine the extent of complex formation (fractional saturation) and these data were plotted as a function of free σ^HrdB^_4_ concentration (Fig. 7*B*) and fitted using a simple binding isotherm. This gave *K_d_* = 7.4 (±1.4) × 10^−7^ m, which indicates a relatively tight binding between the two proteins.

### The SvWhiD-σ^HrdB^_4_ complex depends on the [4Fe-4S] cluster and stabilizes it against O_2_-mediated degradation

Apo*Sv*WhiD was mixed with σ^HrdB^_4_ under the same conditions as [4Fe-4S] *Sv*WhiD at a 1:5 ratio and analyzed by gel filtration (Fig. S9). No evidence for complex formation was observed, indicating that the cluster is required for the *Sv*WhiD-σ^HrdB^_4_ interaction. This is consistent with the data reported for *M. tuberculosis* WhiB1-SigA (12, 15), and with the proposal that the G[V/I]WGG motif is important for mediating protein-protein interactions (11). Indeed, the recent *M. tuberculosis* WhiB1-σ^70^_4_ complex structure showed that a number of residues, including Val-59 and Trp-60 of the conserved motif, as well as Trp-3, Phe-17, and Phe-18 (also conserved in SvWhiD), participate in complex-stabilizing hydrophobic/hydrogen bonding interactions close to the [4Fe-4S] cluster (15). Absence of the cluster would thus be expected to disrupt these interactions and lead to loss of the complex, as found for WhiB1 (12).

Exposure of *Sv*WhiD [4Fe-4S] to aerobic buffer with and without σ^HrdB^_4_ revealed that the presence of σ^HrdB^_4_ protected the WhiD [4Fe-4S] cluster from oxidative degradation. In the absence of σ^HrdB^_4_, *Sv*WhiD [4Fe-4S] was destabilized after 15 min, resulting in precipitation causing increased scattering (Fig. S10). In the presence of σ^HrdB^_4_, a minor decrease in *A*_410 nm_ was observed but the cluster absorbance remained largely stable for more than 1 h. Given the lack of change in the CD upon complex formation (Fig. 1*B*), the protective effect is likely to arise from increased stability of the protein and/or limited accessibility of O_2_ to the cluster. We note that the *M. tuberculosis* WhiB1-σ^70^_4_ complex structure revealed a solvent-inaccessible [4Fe-4S] cluster (15).

### NO-mediated dissociation of the SvWhiD-σ^HrdB^_4_ complex

**Figure 8.**
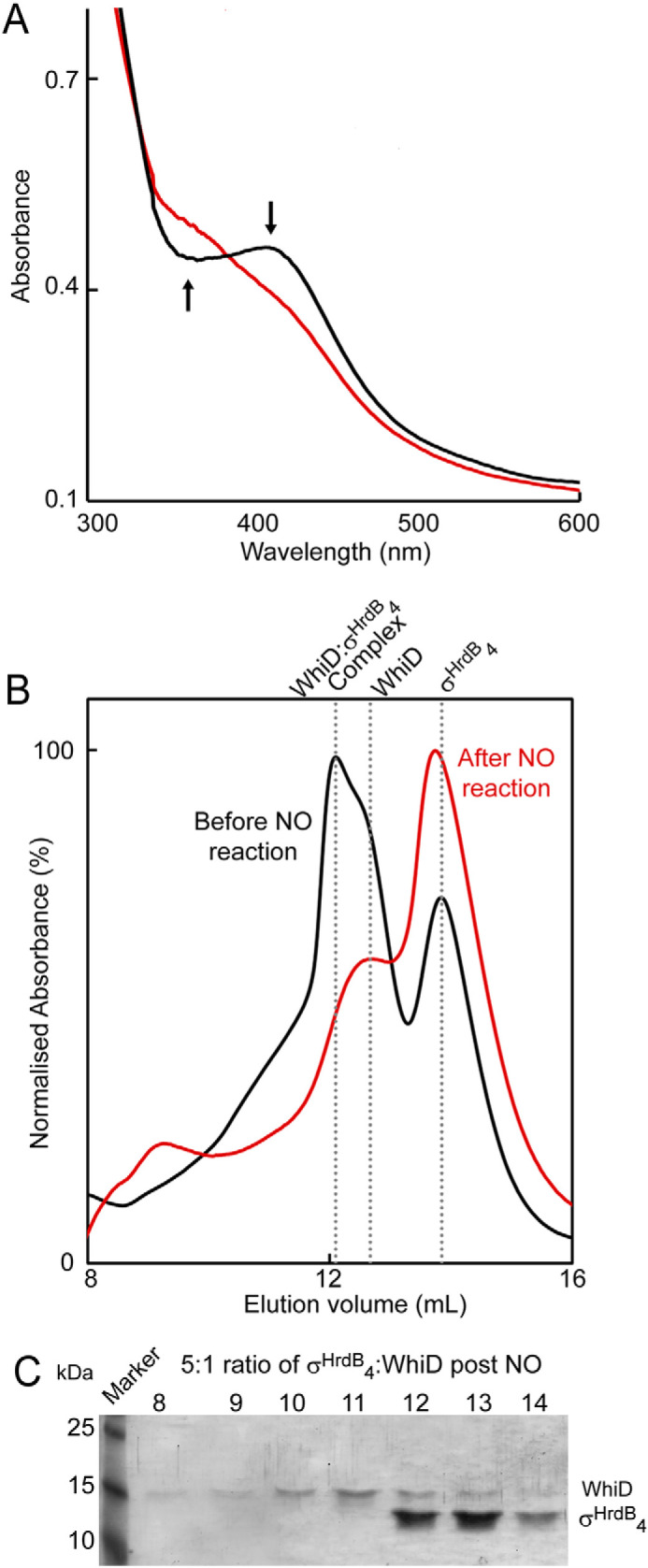
**Reaction of the *Sv*WhiD-σ^HrdB^_4_ complex with NO results in dissociation.** *A*, absorbance spectra of WhiD-σ^HrdB^_4_ complex before (*black line*) and after (*red line*) addition of excess NO (20 NO per cluster). *Arrows* indicate the direction of absorbance change. *B*, gel filtration elution profiles (*A*_280 nm_) of a 5:1 mixture of σ^HrdB^_4_-WhiD before (*black line*) and after (*red line*) reaction with excess NO. *C*, fractions were resolved by SDS-PAGE and visualized by silver staining. *Sv*WhiD (46 μm) and σ^HrdB^_4_ (250 μm) were in 50 mm Tris, 300 mm NaCl, pH 8.

Previous studies of Wbl proteins have demonstrated the sensitivity of the *Sv*Wbl [4Fe-4S] cluster to NO, suggesting that some Wbl proteins may function as NO sensors (17, 19, 21–23). Reaction of the *Sv*WhiD-σ^HrdB^_4_ complex with 20 NO per cluster resulted in the nitrosylation of the [4Fe-4S] cluster (Fig. 8*A*) and dissociation of the complex, as evidenced by gel filtration and SDS-PAGE analysis of the elution fractions (Fig. 8, *B* and *C*).

**Figure 9.**
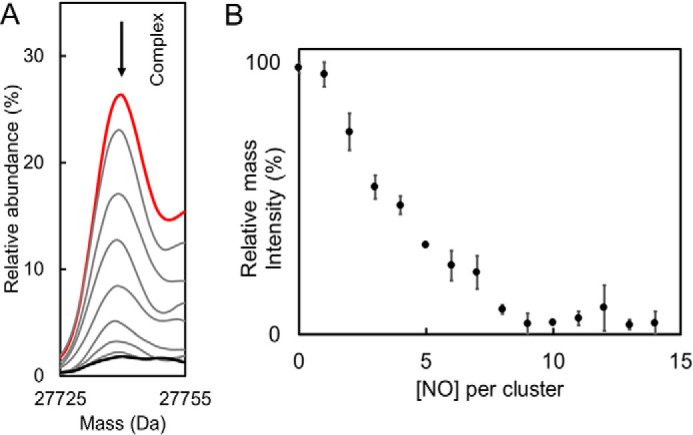
**Nondenaturing mass spectrometric studies of reaction of *Sv*WhiD-σ^HrdB^_4_ complex with NO.** *A*, deconvoluted mass spectra showing the region corresponding to the *Sv*WhiD-σ^HrdB^_4_ complex before (*red spectrum*) and during a titration with NO (up to 14 NO per cluster, *black spectrum*) followed under nondenaturing ESI-MS conditions. The *arrow* shows the trend of the peak during the titration with NO. *B*, plot of percentage relative intensity of *Sv*WhiD-σ^HrdB^_4_ complex as a function of NO per cluster. *Error bars* represent S.E. from *n* = 2 experiments. *Sv*WhiD (10 μm) with σ^HrdB^_4_ in 1:1 excess were in 250 mm ammonium acetate, pH 7.2.

The effect of NO on the *Sv*WhiD-σ^HrdB^_4_ complex was also investigated using nondenaturing MS (12, 22). Fig. 9*A* shows deconvoluted mass spectra in the region corresponding to the complex. The data show clearly the loss of the complex as NO was added, and a plot of intensity as a function of NO per cluster (Fig. 9*B*) indicates that the complex was lost entirely after addition of ∼8 NO molecules per cluster. The form of the plot is similar to that observed for the loss of [4Fe-4S] *Sv*WhiD upon reaction with NO (Fig. 3), suggesting that the same process (*i.e.* reaction of NO with the cluster) is controlling both the loss of the cluster and dissociation of the complex. Again, the plot is not linear, suggesting that the reaction is not fully concerted.

**Figure 10.**
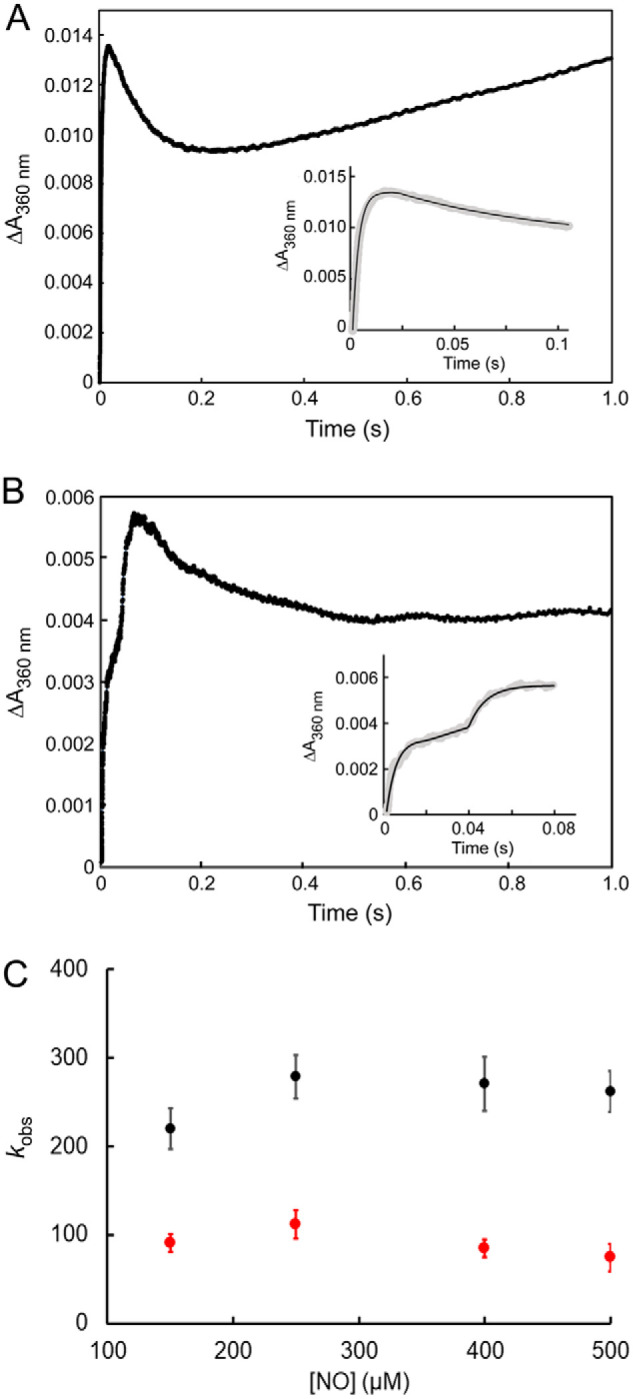
**Stopped-flow kinetic studies of the nitrosylation of the [4Fe-4S] cluster of *Sv*WhiD alone and in complex with σ^HrdB^_4_.** *A*, measurement of absorbance at 360 nm following addition of a 50-fold excess (over [4Fe-4S] cluster) of NO to *Sv*WhiD. *B*, as in (*A*) but with *Sv*WhiD in complex with σ^HrdB^_4_ (with σ^HrdB^_4_ in 4:1 excess). *Insets* show the first 100 and 85 ms in the reaction for *Sv*WhiD and *Sv*WhiD-σ^HrdB^_4_, respectively. *Solid lines* show fits of the observed phases with exponential functions. Experiments were performed with *Sv*WhiD (10 μm in [4Fe-4S]) in 50 mm Tris, 300 mm NaCl, pH 7.2 at 25 °C. *C*, plots of observed rate constants for the first (*black circles*) and second (*red circles*) phases of ΔA_360 nm_ following addition of varying concentrations of NO to *Sv*WhiD-σ^HrdB^_4_. For both, a zero order dependence on NO was observed, indicating that the rate-limiting step of these reactions does not involve NO. Data represent four technical replicates. *Error bars* represent S.E.

Stopped-flow kinetic measurements were performed to determine whether the rate and mechanism of the reaction of the *Sv*WhiD cluster is affected by the presence of σ^HrdB^_4_. Kinetic measurements of reaction of *Sv*WhiD with NO in the absence of σ^HrdB^_4_ were carried out first to determine whether this Wbl protein behaves similarly to those previously characterized: *Sc*WhiD and *M. tuberculosis* WhiB1 (19). For these proteins, kinetic data were consistent with a four-step mechanism of reaction of the Wbl [4Fe-4S] cluster with NO, with three of these steps detectable at 360 nm (19). For *Sv*WhiD, it was immediately apparent that a full kinetic analysis would not be possible because of the instability of the protein during the nitrosylation reaction, with precipitation occurring, as noted above. However, this did not begin to occur until after 500 ms, enabling measurement of the early part of the reaction, Fig. 10*A*. The data revealed the presence of two early phases, which have a similar form to those reported for other Wbl proteins. Plots of the observed apparent first order rate constants (*k*_obs_) against NO concentration were linear for the two phases detected (Fig. S11), indicating two sequential NO reactions that are each first order with respect to NO. The derived rate constants are consistent with those reported for the equivalent phases of nitrosylation of *Sc*WhiD and *M. tuberculosis* WhiB1 (kinetic data are summarized in Table 1). Thus, we conclude that nitrosylation of *Sv*WhiD most likely occurs via a mechanism that is similar to that of other Wbl proteins.

**Table 1:** **Kinetic rate constants for the initial phases of [4Fe-4S] Wbl proteins reactivity with NO**

Phase	Rate constant (m^−1^ s^−1^)		
	*Sv*WhiD	*Sc*WhiD	*Mt*WhiB1
1	(7.37 ± 0.18) × 10^5^	(6.50 ± 0.18) × 10^5^	(4.40 ± 0.44) × 10^5^
2	(2.54 ± 0.001) × 10^4^	(2.13 ± 0.12) × 10^4^	(1.38 ± 0.04) × 10^4^

Stopped-flow measurements were then carried out for the *Sv*WhiD-σ^HrdB^_4_ complex (with a 4-fold excess of σ^HrdB^_4_). Some significant differences were observed. The first phase observed for *Sv*WhiD alone was found to correspond to two consecutive phases in the reaction of the complex with NO, which together occurred over a longer time period (Fig. 10*B*), indicating that [4Fe-4S] *Sv*WhiD is protected to some degree from reaction with NO. Subsequent to this, a phase resembling the second phase of the reaction of *Sv*WhiD alone was observed but kinetic analysis of it was precluded because of precipitation that caused a rising absorbance baseline. Thus, kinetic analyses were focused on the initial phases.

Experiments in which the concentration of NO was varied revealed that the rate of these two initial phases was essentially independent of the NO concentration (Fig. 10*C*), demonstrating that the rate-limiting step in the reaction of the *Sv*WhiD-σ^HrdB^_4_ complex with NO does not involve NO. This is in direct contrast to the reaction of [4Fe-4S] *Sv*WhiD alone with NO, for which a first-order relationship was observed. One possible explanation is that reaction of the cluster with NO cannot occur in the complex in its principal conformation and is thus dependent on a reversible conformational change or dissociation event in order to occur. The data indicate that this initial conformational change/dissociation occurs with a rate constant of ∼100 s^−1^ and is the rate-limiting step of the reaction with NO. The fact that the second phase also appears to be independent of NO concentration suggests that a second conformational change, that could be dependent on the first reaction with NO, is necessary in order for further reaction with NO to occur, and that this is rate-limiting for the second phase of the nitrosylation reaction. These data are consistent with the buried nature of the [4Fe-4S] cluster of WhiB1 in complex with σ^70^_4_ (15), which suggests that an opening up of the structure may be needed for reaction to occur.

## Conclusions

WhiD from *S. venezuelae*, like other Wbl proteins, binds a [4Fe-4S] cluster. Unusually, however, it exists in a monomer-dimer equilibrium, with the monomer-monomer interaction dependent on the C-terminal part of the protein, which is the only part of the protein that is different from the previously characterized monomeric *S. coelicolor* WhiD protein. The significance of dimerization is unclear, but it is interesting to note that the C-terminal extension, which is predicted to form a helix, is conserved in a range of other WhiD homologues.

*Sv*WhiD forms a tight (*K_d_* < 1 μm) and specific complex with the principal sigma factor in Streptomyces, σ^HrdB^. Although interactions between *M. tuberculosis* Wbl proteins and the principal sigma factor in *Mycobacterium* (SigA) have been widely reported, this is the first demonstration that a Wbl protein in *Streptomyces* functions similarly. Thus, the ability to interact with the principal sigma factor is likely to be a conserved feature of Wbl function throughout the actinomycetes. In *Streptomyces* and *Corynebacteria* the ability of the Wbl protein WhiB to function as a transcription factor depends upon a direct interaction with the unrelated transcription factor WhiA. Given our growing understanding of Wbl-sigma interactions, it is possible that a tripartite complex of σ^HrdB^, WhiB and WhiA regulates gene expression. One possible explanation for such a requirement could be the lack of a distinct DNA-binding motif in Wbl proteins. WhiB, like most Wbl family members, only carries a series of C-terminal, positively charged amino acid residues that may increase affinity for DNA (the exception is WblC/WhiB7, which binds DNA via a C-terminal AT-hook motif). Thus, the ability of WhiB to function as a transcription factor is mediated via WhiA, which carries an HTH motif. The interaction between the Wbl-family member WhiB and another transcription factor, WhiA, raises the possibility that other Wbl proteins function via interactions with proteins other than the principal sigma factor. Work toward resolving this question is ongoing.

The interaction between Wbls and the principal sigma factor depends on the [4Fe-4S] cluster, shown both here in *Streptomyces* between WhiD and σ^HrdB^ and in *Mycobacteria* between WhiB1 and SigA. Thus, reaction with O_2_ or NO, which results in cluster degradation, leads to disassembly of the complex, illustrating a likely mechanism by which environmental signals could be transduced to regulatory responses. In the case of reaction with NO, this is a multistep process as previously described for other Wbl proteins. Complex formation significantly protects the cluster from O_2_-mediated degradation.

The [4Fe-4S] cluster is also protected to some degree in the complex from reaction with NO, and it is reasonable to suggest that the accessibility of NO to the cluster is impaired because of the interaction of WhiD with σ^HrdB^. However, reaction still occurs, most likely because of conformational flexibility or reversible dissociation that occurs more slowly than the initial reaction of the unhindered cluster with NO. We note that the overall effect, however, is that the reactions with O_2_ and NO are kinetically even more distinct than for [4Fe-4S] *Sv*WhiD alone, such that the *Sv*WhiD-σ^HrdB^ complex is optimally arranged to distinguish between O_2_ and NO.

## Experimental procedures

### Overexpression and purification of SvWhiD

Bacterial strains, plasmids and oligonucleotides used in this study are listed in Table S1. A codon-optimized gene was synthesized (GenScript) and subsequently ligated into pET28a using *Ndel* and HindIII sites, generating pMSW1, for the expression of N-terminally His_6_-tagged *S. venezuelae* WhiD in *Escherichia coli*. The protein was overproduced in 5 liters (8 × 1 liter flasks) aerobically grown *E. coli* BL21 (DE3) cultures in LB containing 50 μg/ml kanamycin. Cultures were grown at 37 °C with shaking at 200 rpm until *A*_600_ reached 0.6–0.8, at which point flasks were placed on ice for 18 min. Protein expression was induced with 0.3 mm IPTG and cultures incubated for 50 min at 30 °C, with shaking at 105 rpm. Cultures were then supplemented with 200 μm ammonium ferric citrate and 25 μm l-methionine to promote [Fe-S] cluster incorporation and incubated at 30 °C, 105 rpm for a further 4 h. Cells were harvested by centrifugation at 8000 rpm at 4 °C for 10 min and stored at −80 °C until required.

Unless stated, all purification steps were performed in an anaerobic cabinet (O_2_ < 2 parts/million). Cell pellets were resuspended in buffer A (50 mm Tris, 300 mm NaCl, 25 mm imidazole, pH 7.5) with the addition of lysozyme (300 μg/ml) and PMSF (300 μg/ml). Resuspended cells were removed from the anaerobic cabinet and lysed by sonication on ice under N_2_, twice for 8 min 20 s, 0.2-s bursts, 50% power and immediately returned to the anaerobic cabinet. Lysed cells were centrifuged in air-tight centrifuge tubes outside of the anaerobic cabinet at 40,000 × *g* for 45 min at 1 °C and returned into the anaerobic cabinet.

The supernatant was loaded onto a HiTrap Ni-affinity column, washed with buffer A until A_280 nm_ was <0.1. Bound proteins were eluted with buffer B (50 mm Tris, 300 mm NaCl, 500 mm imidazole, pH 7.5) from 0 to 100% (v/v) over a linear gradient of 10-ml fractions containing WhiD were pooled and desalted using a HiTrap desalting column into buffer C (50 mm Tris, 300 mm NaCl, pH 7.5) and stored in an anaerobic freezer until needed. Total protein concentration was determined using the Bradford (Bio-Rad) (34) or Rose Bengal (35) assays, with BSA as calibration standard. Purity of the protein was checked using SDS-PAGE gel electrophoresis and LC-MS. Cluster concentration was determined by reference to a calibration curve generated from Fe^3+^ solutions prepared from SpectrosoL standard iron solution (36), or by using an absorbance extinction coefficient at 410 nm of 17,500 m ^−1^ cm ^−1^.

A codon-optimized gene encoding a truncated form of WhiD lacking the 33 C-terminal residues was synthesized (GenScript) and subsequently ligated into pET28a using *Ndel* and HindIII sites, for the expression of N-terminally His_6_-tagged *S. venezuelae* truncated WhiD in *E. coli*. The protein was overexpressed and purified as described for full-length *Sv*WhiD. Protein and cluster concentrations were determined as above.

### Overexpression and purification of domain 4 of σ^HrdB^ and σ^HrdD^

Codon-optimized genes for the expression of N-terminally His_6_-tagged σ^HrdB^_4_ and σ^HrdD^_4_ were also synthesized (GenScript) and ligated into pET15b using *Ndel* and BamHI sites, generating pMSW2 and pMSW3. *S. venezuelae* σ^HrdB^_4_ and σ^HrdD^_4_ proteins were overproduced in 5 liters (8 × 1 liter flasks) aerobically grown *E. coli* BL21 (DE3) cultures in LB containing 100 μg/ml ampicillin. Cultures were grown at 37 °C, 200 rpm until *A*_600_ reached 0.6–0.8, at which point overexpression of proteins was induced by the addition of 0.5 mm IPTG. Cultures were incubated further at 37 °C, 200 rpm for 4 h. Cells were harvested by centrifugation at 8000 rpm at 4 °C for 10 min and stored at −80 °C until required. *S. venezuelae* σ^HrdB^_4_ and σ^HrdD^_4_ proteins were purified as described above for WhiD, except that all steps were carried out under aerobic conditions.

### Overexpression and purification of I151A FNR

Aerobic cultures of *E. coli* BL21λDE3 containing pGS2252 (encoding I151A GST-FNR) were grown and protein isolated as described previously (37), except that aerobic conditions were employed to generate the cluster-free form of I151A GST-FNR. I151A FNR was cleaved from the fusion protein using thrombin, as described previously (38).

### Bacterial two-hybrid (BACTH) genomic library construction, screening, and analysis

Construction of genomic BACTH libraries was performed by BIO S&T (Saint-Laurent, Québec, Canada) as described previously (39). To construct the “bait” vector, the *whiD* gene was amplified using the whiD_BACTH_F and whiD_BACTH_R primers and cloned into the pKT25 plasmid digested with XbaI and BamHI to create the plasmid pIJ10921 encoding the T25 domain of adenylate cyclase fused to the N terminus of WhiD (T25-WhiD). The *E. coli* BTH101 strain was transformed with pIJ10921 before electroporation of ∼0.125 μg of the T18C genomic library. Transformations were recovered in SOC medium, washed twice with M63 and then plated onto M63 minimal medium, supplemented with 0.3% lactose, 50 μg/ml Carb, 25 μg/ml Kan, 0.5 mm IPTG, and 40 μg/ml X-gal. Plates were incubated for 5–10 days at 30 °C and colonies were then restreaked onto MacConkey agar supplemented with 1% maltose, 0.5 mm IPTG, 100 μg/ml Carb, and 50 μg/ml Kan, incubating for 2 days at 30 °C. Strongly interacting clones (*cyaA*+), identified by their red color were grown in liquid culture overnight, selecting only for the pUT18C “prey” plasmid (100 μg/ml Carb). DNA was isolated by Miniprep (Qiagen) and sequenced using the pUT18C_F primer.

BACTH vectors encoding the T18 domain of adenylate cyclase fused to the N terminus of σ^HrdB^ and σ^HrdD^ (T18-σ^HrdB^ and T18-σ^HrdD^) were constructed. Full-length *hrdB* and *hrdD* genes were amplified using the primer pairs hrdB_BACTH_F/hrdB_BATCH_R and hrdD_BACTH_F/hrdD_BACTH_R, respectively, and cloned into the pUT18C plasmid digested with XbaI and KpnI to create plasmids pIJ10922 and pIJ10923. Sequences encoding σ^HrdB^ region 4 only and σ^HrdB^ lacking region 4 were amplified using the primer pairs hrdB4_BACTH_F/hrdB4_BACTH_R and hrdBd4_BACTH_F/hrdBd4_BACTH_R, respectively, and similarly cloned to generate plasmids pIJ10925 and pIJ10926. To test interactions between proteins, *E. coli* BTH101 was co-transformed with the appropriate pKT25 and pUT18C fusion plasmids. β-gal assays were conducted as in previous studies (*e.g.* (40, 41)), according to standard methodology (42). Cultures were additionally spotted (7.5 μl) onto M63 minimal medium, supplemented with 0.3% lactose, 50 μg/ml Carb, 25 μg/ml Kan, 0.5 mm IPTG, and 40 μg/ml X-gal.

### Analytical gel filtration

Gel filtration was performed aerobically using a pre-calibrated Superdex 75 10/300 GL column, at room temperature in buffer D (50 mm Tris, 300 mm NaCl, pH 8.0) with a flow rate of 0.5 ml/min. Buffers were purged with N_2_ and kept in an anaerobic glovebox overnight until needed. Mass of proteins was estimated by reference to a calibration curve generated using BSA, bovine erythrocyte carbonic anhydrase, and horse heart cytochrome *c* at 10 mg/ml, 3 mg/ml, and 2 mg/ml, respectively, dissolved in buffer D. Protein fractions were then analyzed by SDS-PAGE gel and visualized by silver stain using standard protocols (ProteoSilver^TM^, Sigma-Aldrich).

### Spectroscopic measurements

UV-visible absorbance measurements were made using a Jasco V550 spectrometer. CD spectra were measured with a Jasco J810 spectropolarimeter. Samples for spectroscopy were in buffer C (50 mM Tris, 300 mM NaCl, pH 7.5).

### Mass spectrometry

*Sv*WhiD and σ^HrdB^_4_ were buffer exchanged into 250 mm ammonium acetate, pH 7.2, inside an anaerobic cabinet using mini-PD10 (GE Healthcare) desalting columns. The concentration of [4Fe-4S] WhiD was determined via absorbance at 410 nm and the concentration of σ^HrdB^_4_ in ammonium acetate was determined using a Bio-Rad dye kit, as described above.

Proteins or protein mixtures were infused directly into the ESI source of a Bruker micrOTOF-QIII mass spectrometer operating in positive ion mode using at 0.3 ml/h using a syringe pump. MS data were acquired over the *m*/*z* range of 1000–3500 continuously for 5 min using Bruker oTOF Control software, with parameters as follows: dry gas flow of 4 liters/min, nebulizer gas pressure 0.8 Bar, dry gas 180 °C, capillary voltage 4500 V, offset 500 V, ion energy 5 eV, collision RF 650 Vpp, collision cell energy 10 eV. Processing and analysis of MS experimental data were carried out using Compass DataAnalysis version 4.1 (Bruker Daltonik, Bremen, Germany), and neutral mass spectra were generated using the ESI Compass version 1.3 Maximum Entropy deconvolution algorithm. Exact masses (± 1 Da) are reported from peak centroids representing the isotope average neutral mass. For apoproteins, exact masses were derived from *m*/*z* spectra, for which peaks correspond to [M + nH]^n+^/n. For [4Fe-4S]^2+^ WhiD, peaks corresponded to [m + [4Fe-4S]^2+^ + (n-2)H]^n+^/n, where m is the molecular mass of the protein, [4Fe-4S] is the mass of the iron-sulfur cluster of 2+ charge, H is the mass of the proton, and n is the total charge (22, 24, 43).

For NONOate experiments, the reaction syringe was maintained at 25 °C. The mass spectrometer was calibrated with ESI-low concentration tuning mix in positive ion mode (Agilent Technologies). For ESI-MS NO titration experiments, MS intensity data were processed to generate relative abundance plots of ion counts for the [4Fe-4S] form as a fraction of the total ion count because of [4Fe-4S]-bound and apo forms. This permitted changes in relative abundance to be followed without distortions because of variations in ionization efficiency that normally occur across a data collection run. For LC-MS, proteins were diluted in an aqueous mixture of 2% (v/v) acetonitrile and 0.1% (v/v) formic acid and analyzed as described previously (21).

For the determination of the dissociation constant for the WhiD-σ^HrdB^_4_ complex, FNR I151A (37) was used as an internal standard. Solutions of equimolar WhiD and FNR (3 μm final concentrations), pre-exchanged into 250 mm ammonium acetate, pH 7.2, were mixed with increasing concentrations of σ^HrdB^_4_ in the same buffer to give molar ratios of σ^HrdB^_4_-WhiD between 0 and 7. Mixed samples were incubated for 5 min before loading into a 500 μl gastight syringe and infused directly into the mass spectrometer operating in positive mode. Parameters were as described above for nondenaturing experiments. Each data point was collected over 2 min average (three replicas) and deconvoluted over the range of 10 to 30 kDa. Ion counts for the WhiD-σ^HrdB^_4_ complex were compared with the combined ion counts for FNR and complex. Data were then expressed as fractional saturation and fitted using a simple binding isotherm from which a *K_d_* for the complex was obtained.

### Nitric oxide reactivity experiments

For nondenaturing ESI-MS experiments, NO donor DEA-NONOate (Sigma-Aldrich) solutions were prepared immediately before use, in 4 °C ammonium acetate buffer and quantified by absorbance at 250 nm (ϵ = 6500 m^−1^ cm^−1^). The half-life of DEA-NONOate at 25 °C in 250 mm ammonium acetate buffer, pH 7.2, was determined as *t*_½_ ∼7 min, yielding overall 1.5 NO molecules per NONOate (22). DEA-NONOate was added directly to WhiD samples to give a specific ratio of NO to [4Fe-4S] cluster of 20 over the course of 50 min. Spectra were averaged over 2.5 min to obtain data at intervals of 1.1 NO per cluster. For UV-visible absorbance experiments, PROLI-NONOate (Cayman Chemicals) solutions were prepared in 50 mm NaOH and quantified by absorbance at 252 nm (ϵ = 8400 m^−1^ cm^−1^). PROLI-NONOate was titrated into the sample and incubated for 5 min at ambient temperature prior to measurement, to allow full NO release from NONOate (*t*_½_ ∼2 s).

For UV-visible stopped-flow experiments, a Pro-Data–upgraded Applied Photophysics Bio-Sequential DX.17 MV spectrophotometer was used, with a 1-cm path length cell. Absorption changes were detected at 360 nm. Experiments were carried out in 50 mm Tris 300 mm NaCl, pH 7.2) using gastight syringes (Hamilton) at 25 °C. Prior to use, the stopped-flow instrument was flushed with ∼50 ml of anaerobic buffer. Solutions of *Sv*WhiD-σ^HrdB^_4_ complex were prepared at a 4 to 1 excess of σ^HrdB^_4_ to *Sv*WhiD. All solutions used for stopped-flow experiments were stored and manipulated inside an anaerobic cabinet (Belle Technology). NO solutions were prepared using PROLI-NONOate, as described above. Final traces are averages of 10 individual traces. Fitting of kinetic data were performed using OriginPro8 (OriginLabs).

### Data availability

All of the data are contained within the main paper and supporting information. In addition, *x*,*y* data for figure plots and original gel images are available at Open Science Framework, doi: 10.17605/OSF.IO/HVC9R.

## Author contributions

M. Y. Y. S. and M. J. Bush data curation; M. Y. Y. S. and M. J. Bush formal analysis; M. Y. Y. S., M. J. Bush, and N. E. L. B. investigation; M. Y. Y. S. and M. J. Bush visualization; M. Y. Y. S. and J. C. C. methodology; M. Y. Y. S., M. J. Bush, and N. E. L. B. writing-original draft; M. J. Bush, M. J. Buttner, and N. E. L. B. conceptualization; J. C. C., M. J. Buttner, and N. E. L. B. supervision; J. C. C., M. J. Buttner, and N. E. L. B. writing-review and editing; M. J. Buttner and N. E. L. B. project administration; N. E. L. B. funding acquisition.

## Supplementary Material

Supporting Information

## Footnotes

2 The abbreviations used are:

FNR: fumarate and nitrate reduction
IPTG: isopropyl 1-thio-β-d-galactopyranoside.
