# Supplementary material for: Interaction of the Streptomyces Wbl protein WhiD with the principal sigma factor σHrdB depends on the WhiD [4Fe-4S] cluster
Source: J Biol Chem. 2020 Apr 17;295(28):9752–65. doi: 10.1074/jbc.RA120.012708 (PMC7363131; doi:10.1074/jbc.RA120.012708)
Supplement: Supporting Information [file supp_295_28_9752__index.html]

Interaction of the Streptomyces Wbl protein WhiD with the principal sigma factor σHrdB depends on the WhiD [4Fe-4S] cluster — Iron-sulfur cluster dependent binding of WhiD to σHrdB — Interaction of the Streptomyces Wbl protein WhiD with the principal sigma factor σHrdB depends on the WhiD [4Fe-4S] cluster — Iron-sulfur cluster–dependent binding of WhiD to σHrdB — Supporting Information 

# Interaction of the *Streptomyces* Wbl protein WhiD with the principal sigma factor σHrdB depends on the WhiD [4Fe-4S] cluster

## Supporting Information

- Supporting Information (to be published online) - Supporting information
