# Supplementary material for: Interaction of the Streptomyces Wbl protein WhiD with the principal sigma factor σHrdB depends on the WhiD [4Fe-4S] cluster
Source: J Biol Chem. 2020 Apr 17;295(28):9752–65. doi: 10.1074/jbc.RA120.012708 (PMC7363131; doi:10.1074/jbc.RA120.012708)
Supplement: Supporting Information [file supp_RA120.012708_158310_2_supp_511652_q8tkwh.pdf]

Supporting Information

Interaction of the *Streptomyces* Wbl protein WhiD with the principal sigma factor σ^{HrdB} depends on the WhiD [4Fe-4S] cluster

Melissa Y. Y. Stewart, Matthew J. Bush, Jason C. Crack, Mark J. Buttner and Nick E. Le Brun

Contents

Table S1. Strains, plasmids and oligonucleotides
Figure S1. Mass analysis and sequence alignment of *Sv*WhiD
Figure S2. Gel filtration analysis of truncated *Sv*WhiD
Figure S3. Spectroscopic and spectrometric analysis of truncated *Sv*WhiD
Figure S4. The C-terminal extension of *Sv*WhiD is found in WhiD homologues from other *Streptomyces* species
Figure S5. Reaction of *Sv*WhiD with NO
Figure S6. T18C-σ^{HrdB} fusion start points for the five positive σ^{HrdB} clones identified from a shotgun *S. venezuelae* sonicated DNA genomic library using T25-WhiD as bait.
Figure S7. Amino acid sequence of $\sigma^{HrdB}{}_4$ and $\sigma^{HrdD}{}_4$ used in studies of interactions with *Sv*WhiD
Figure S8. Non-denaturing mass spectrometry of mixtures containing [4Fe-4S] *Sv*WhiD and $\sigma^{HrdB}{}_4$
Figure S9. Apo-*Sv*WhiD does not bind to domain 4 of σ^{HrdB}, the principal sigma factor of *S. venezuelae*
Figure S10. Sensitivity of *Sv*WhiD to O_2 is modulated by complex formation with $\sigma^{HrdB}{}_4$
Figure. S11. Stopped-flow kinetic studies of the nitrosylation of the [4Fe-4S] cluster of *Sv*WhiD
Supporting references

Table S1. Strains, plasmids and oligonucleotides.

	Description	Reference
**Strains**		
BTH101	F– *cya-99 araD139 galE15 galK16 rpsL1 (Strr) hsdR2 mcrA1 mcrB1*	30
**Plasmids**		
pkT25		30
pUT18C		30
pIJ10921	*whiD* cloned into pKT25	This work
pIJ10922	*hrdB* cloned into pUT18C	This work
pIJ10923	*hrdD* cloned into pUT18C	This work
pIJ10925	*hrdB4* cloned into pUT18C	This work
pIJ10926	*hrdBΔ4* cloned into pUT18C	This work
pET28a	Expression vector with N-terminal hexahistidine affinity tag	Novagen
pET15b	Expression vector with N-terminal hexahistidine affinity tag	Novagen
pMSW1	*whiD* cloned into pET28a	This work
pMSW2	*hrdB* (encoding domain 4) cloned into pET15b	This work
pMSW3	*hrdD* (encoding domain 4) cloned into pET15b	This work
**Oligonucleotides**		
whiD_BACTH_F	GCTCTAGAGATGGCAGATTTCTCCCGCCTT	
whiD_BACTH_R	CGCGGATCCTCCATGCGCTCCACAATCGGAC	
hrdB_BACTH_F	GCTCTAGAGGTGACGGCCGAGGAAGGAATA	
hrdB_BACTH_R	CCGGTACCCGGTCGAGGTAGTCGCGCAG	
hrdD_BACTH_F	GCTCTAGAGATGGCAACCCGTGCCGTCG	
hrdD_BACTH_R	CCGGTACCCGGGCCACCGCGTCGAAGCC	
hrdB4_BACTH_F	GCTCTAGAGATGCCGGCCGACGCGGTGAGC	
hrdB4_BACTH_R	CCGGTACCCGGTCGAGGTAGTCGCGCAGCACCTGC	
hrdBd4_BACTH_F	GCTCTAGAGATGACGGCCGAGGAAGGAATACAGCA	
hrdBd4_BACTH_R	CCGGTACCCGAACGACCGCCTCGGAGTCCTC	
pUT18C_F	GTGCCGAGCGGACGTTCGA	

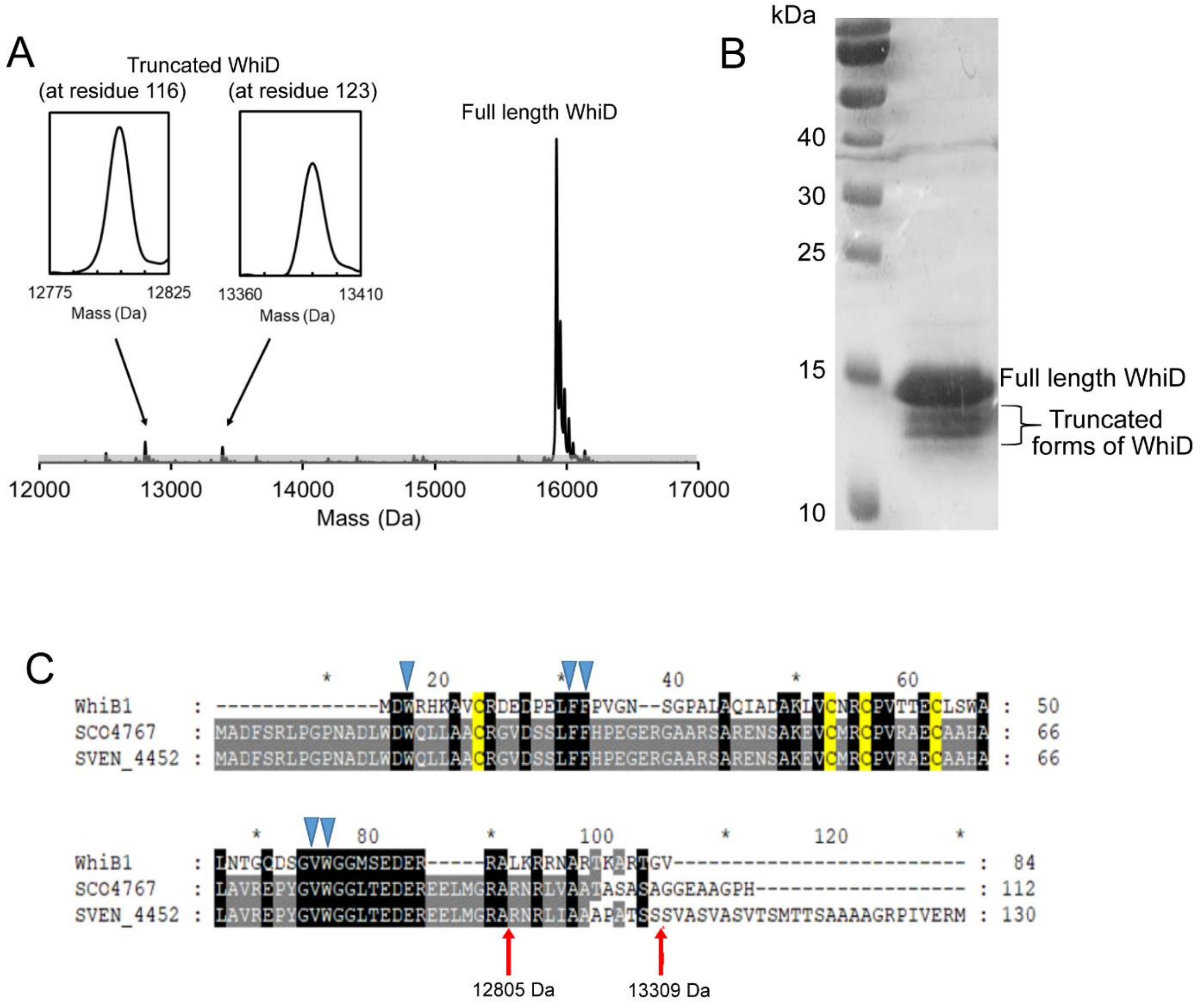

Figure S1. Mass analysis and sequence alignment of *Sv*WhiD. (**A**) Deconvoluted LC-MS spectrum of as isolated *Sv*WhiD, revealing the major species as *Sv*WhiD lacking its N-terminal Met residue. Minor components of two truncated forms are apparent. Inset are spectra of the truncated form plotted on an expanded mass scale. *Sv*WhiD (10 µM) was in an aqueous mixture of 2% (v/v) acetonitrile, 0.1% (v/v) formic acid. (**B**) SDS-PAGE analysis of as isolated *Sv*WhiD, revealing full length and truncated forms. (**C**) Amino acid sequence alignment of *Sv*WhiD (SVEN_4452) with *S. coelicolor* WhiD (SCO4767) and *M. tuberculosis* WhiB1. Conserved Cys residues are highlighted in yellow. Residues shown to be important for the interaction of WhiB1 with domain 4 of SigA are indicated by blue triangles. Positions of truncation for smaller versions of *Sv*WhiD are indicated.

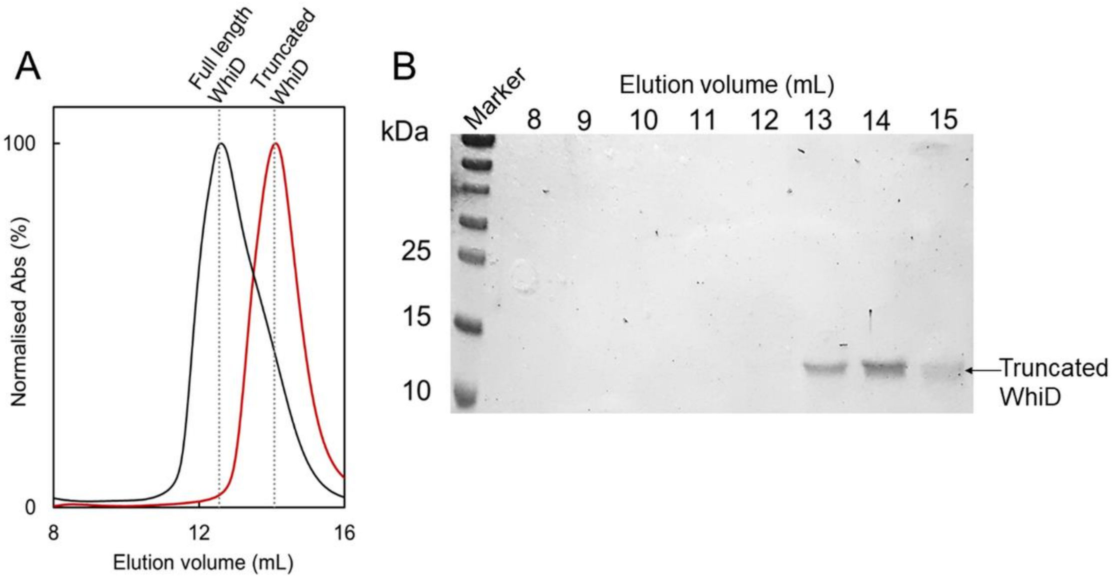

Figure S2. Gel filtration analysis of truncated *Sv*WhiD. (**A**) Gel filtration elution profiles ($A_{280\ nm}$) of truncated [4Fe-4S] WhiD (red line). The elution profile for full length [4Fe-4S] WhiD is shown for comparison (black line). (**B**) SDS-PAGE analysis of elution fractions for truncated *Sv*WhiD, spanning the elution volume 8-15 mL. Truncated *Sv*WhiD (32 µM) was in 50 mM Tris, 300 mM NaCl, pH 8.

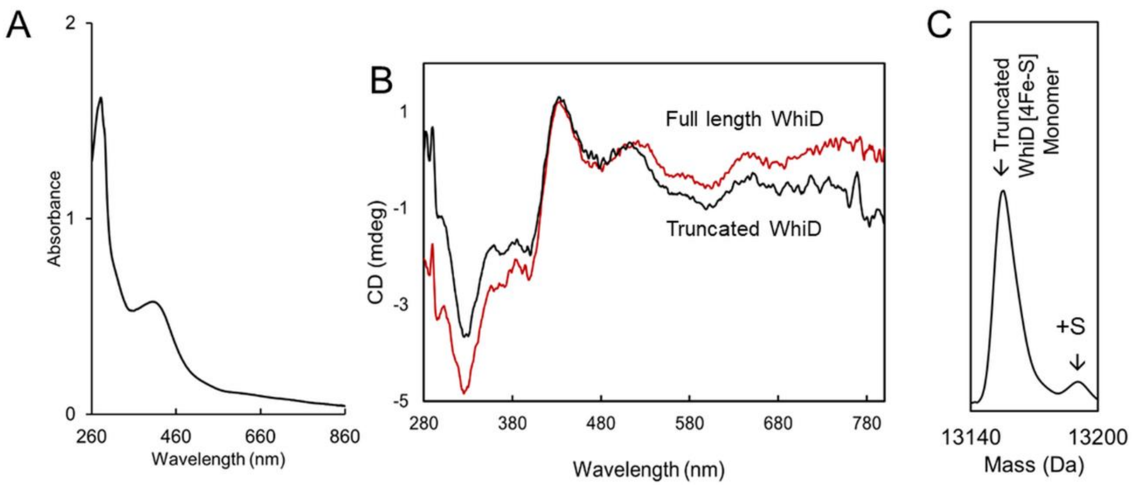

Figure S3. Gel filtration analysis of truncated *Sv*WhiD. (**A**) UV-visible absorbance and (**B**) CD spectroscopic characterisation of truncated WhiD. In (B) spectra of truncated and full length *Sv*WhiD are shown in black and red, respectively. (**C**) Deconvoluted non-denaturing ESI-MS. The spectrum features a major peak due to truncated [4Fe-4S] WhiD. A possible sulfur adduct is indicated. For spectroscopy experiments, truncated *Sv*WhiD (32 μM) was in 50 mM Tris, 300 mM NaCl, pH 7.2. For non-denaturing ESI-MS, *Sv*WhiD (15 μM) was in 250 mM ammonium acetate, pH 7.2.

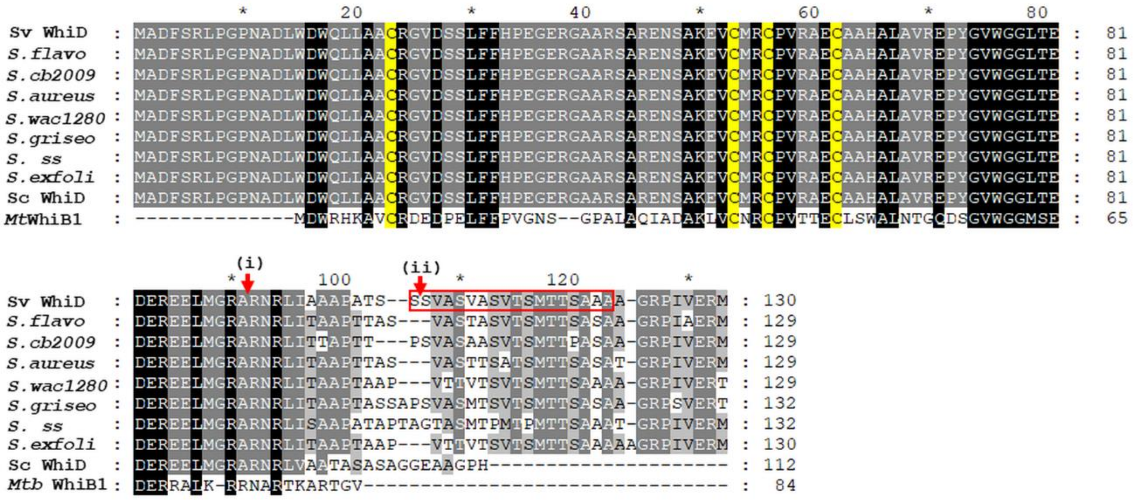

Figure S4. The C-terminal extension of *Sv*WhiD is found in WhiD homologues from other *Streptomyces* species. Alignment of *Sv*WhiD with putative WhiD sequences from *Streptomyces flavochromogenes*, *Streptomyces sp*. CB02009, *Streptomyces aureus*, *Streptomyces* sp. WAC01280, *Streptomyces griseoluteus*, *Streptomyces* sp. SS, and *Streptomyces exfoliates*. *Streptomyces coelicolor* WhiD (*Sc*WhiD) and *Mycobacterium tuberculosis* WhiB1 (Mtb WhiB1) are shown for comparison. Conserved Cys residues are highlighted in yellow. C-terminally truncated forms of *Sv*WhiD as indicated by arrows correspond to i) 12,805 Da and ii) 13,309 Da. Putative dimerization helix is boxed in red.

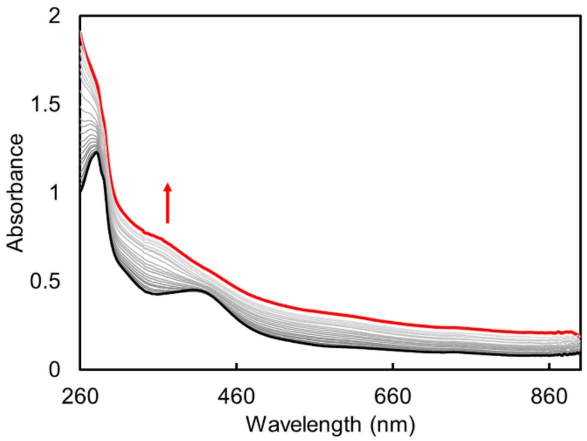

Figure S5. Reaction of *Sv*WhiD with NO. UV-visible absorbance titration of [4Fe-4S] *Sv*WhiD with up to 20 NO per cluster. Black and red spectra correspond to the first and final spectra, respectively, with intervening spectra in grey. The arrow indicates the direction of change due to a combination of absorbance changes and scattering due to mild precipitation of the protein. WhiD (21 µM) was in 50 mM Tris, 300 mM NaCl, pH 7.2.

```
CLONE 1        ------------------------------------------------------------------------------------------
CLONE 2    390 -----------------------------IRQAITRAMADQARTIRIPVHMVEVINKLARVQRQMLQDLGREPTPEELAKELDMTPEKVI 450
CLONE 3    390 -----------------------------IRQAITRAMADQARTIRIPVHMVEVINKLARVQRQMLQDLGREPTPEELAKELDMTPEKVI 450
CLONE 4    368 -------IRAVEKFDYTKGYKFSTYATWWIRQAITRAMADQARTIRIPVHMVEVINKLARVQRQMLQDLGREPTPEELAKELDMTPEKVI 450
CLONE 5    368 -------IRAVEKFDYTKGYKFSTYATWWIRQAITRAMADQARTIRIPVHMVEVINKLARVQRQMLQDLGREPTPEELAKELDMTPEKVI 450
HrdB       361 QEGNLGLIRAVEKFDYTKGYKFSTYATWWIRQAITRAMADQARTIRIPVHMVEVINKLARVQRQMLQDLGREPTPEELAKELDMTPEKVI 450
                      *                     *

CLONE 1    488 -------------------------------------DAVSFTLLQEQLHSVLDTLSEREAGVVSMRFGLTDGQPKTLDEIGKVYGVTRE 540
CLONE 2    451 EVQKYGREPISLHTPLGEDGDSEFGDLIEDSEAVVPADAVSFTLLQEQLHSVLDTLSEREAGVVSMRFGLTDGQPKTLDEIGKVYGVTRE 540
CLONE 3    451 EVQKYGREPISLHTPLGEDGDSEFGDLIEDSEAVVPADAVSFTLLQEQLHSVLDTLSEREAGVVSMRFGLTDGQPKTLDEIGKVYGVTRE 540
CLONE 4    451 EVQKYGREPISLHTPLGEDGDSEFGDLIEDSEAVVPADAVSFTLLQEQLHSVLDTLSEREAGVVSMRFGLTDGQPKTLDEIGKVYGVTRE 540
CLONE 5    451 EVQKYGREPISLHTPLGEDGDSEFGDLIEDSEAVVPADAVSFTLLQEQLHSVLDTLSEREAGVVSMRFGLTDGQPKTLDEIGKVYGVTRE 540
HrdB       451 EVQKYGREPISLHTPLGEDGDSEFGDLIEDSEAVVPADAVSFTLLQEQLHSVLDTLSEREAGVVSMRFGLTDGQPKTLDEIGKVYGVTRE 540
                                                    *

CLONE 1    541 RIRQIESKTMSKLRHPSRSQVLRDYLD 567
CLONE 2    541 RIRQIESKTMSKLRHPSRSQVLRDYLD 567
CLONE 3    541 RIRQIESKTMSKLRHPSRSQVLRDYLD 567
CLONE 4    541 RIRQIESKTMSKLRHPSRSQVLRDYLD 567
CLONE 5    541 RIRQIESKTMSKLRHPSRSQVLRDYLD 567
HrdB       541 RIRQIESKTMSKLRHPSRSQVLRDYLD 567
```

Figure S6. T18C-σ^{HrdB} fusion start points for the five positive σ^{HrdB} clones identified from a shotgun *S. venezuelae* sonicated DNA genomic library using T25-WhiD as bait. The sequences of the five clones are aligned against the C-terminal protein sequence of σ^{HrdB} with asterisks indicating the fusion start points. Domain 4 (σ$^{HrdB}_{4}$) is underlined.

$\sigma^{HrdB}{}_4$:
MGSSHHHHHHSSGLVPRGSHMPADAVSFTLLQEQLHSVLDTLSEREAGVVSMRFGLTDGQPKTLDEIG
KVYGVTRERIRQIESKTMSKLRHPSRSQVLRDYLD

$\sigma^{HrdD}{}_4$:
MGSSHHHHHHSSGLVPRGSHMPEQSVLTLLRSEELDDLIDKLDHRTASIIRMRYGIEDGRERTLTEVG
KEHGLTRERIRQIEKHALLELKKMAHDTGFDAVA

Figure S7. Amino acid sequence of $\sigma^{HrdB}{}_4$ and **$\sigma^{HrdD}{}_4$ used in studies of interactions with *Sv*WhiD.** The tag sequence is indicated in green. For both proteins, the N terminal methionine was cleaved during over-expression.

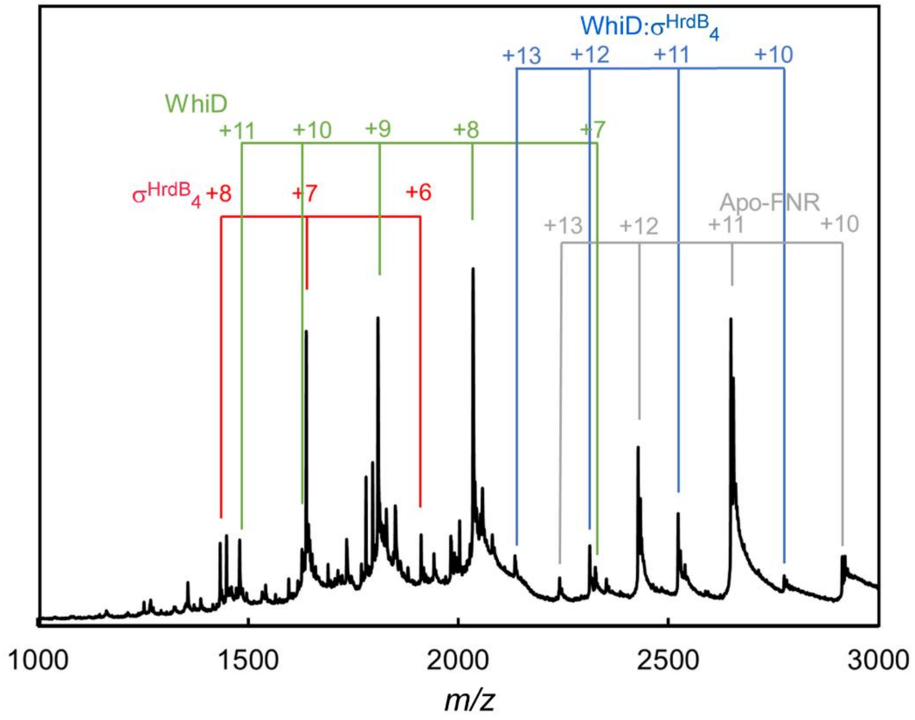

Figure S8. Non-denaturing mass spectrometry of mixtures containing [4Fe-4S] *Sv*WhiD and $\sigma^{HrdB}{}_4$. *m/z* spectra of a solution containing *Sv*WhiD and $\sigma^{HrdB}{}_4$ (present in 4-fold excess). Ions due to unbound proteins, and the *Sv*WhiD-$\sigma^{HrdB}{}_4$ complex are as indicated. Ions due to apo- I151A FNR are also present, as this species was used as an internal intensity standard for measuring changes in the concentration of the complex. *Sv*WhiD (16 µM in cluster) and $\sigma^{HrdB}{}_4$ were in 250 mM ammonium acetate, pH 7.2.

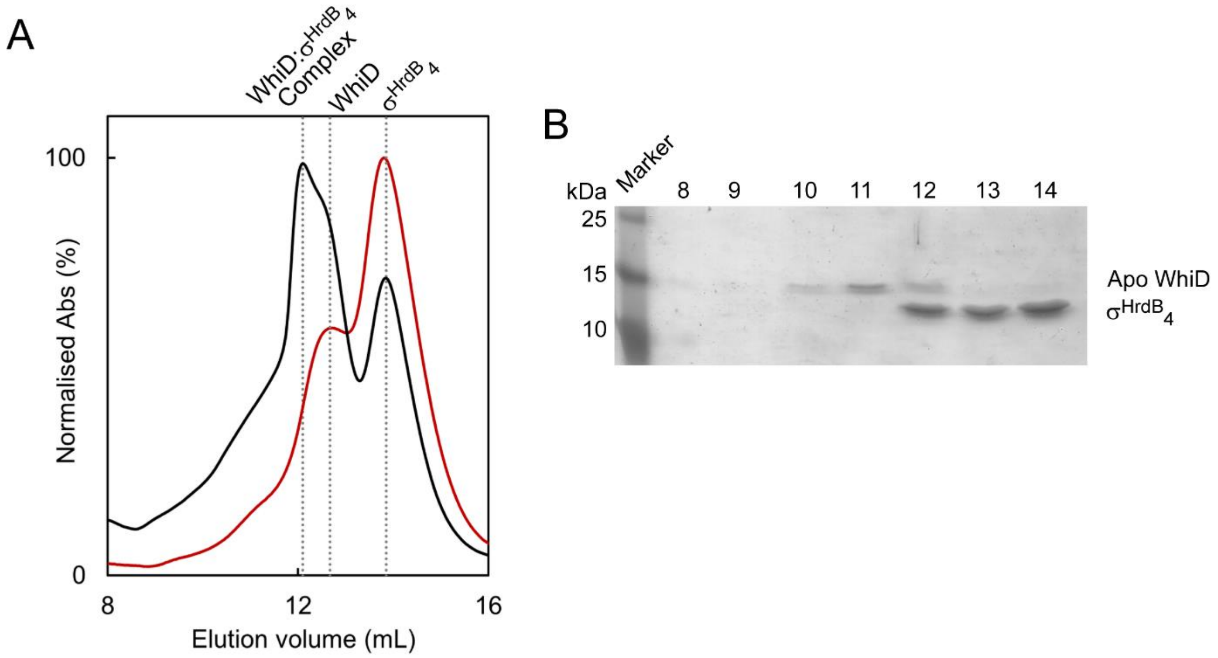

Figure S9. Apo-*Sv*WhiD does not bind to domain 4 of σ^{HrdB}, the principal sigma factor of *S. venezuelae*. (**A**) Gel filtration elution profiles ($A_{280\ nm}$) of a mixture of apo-*Sv*WhiD and $\sigma^{HrdB}{}_4$ in a 1:5 ratio (red line). The elution profile of a mixture of cluster-containing *Sv*WhiD and $\sigma^{HrdB}{}_4$ in a 1:5 ratio is shown for comparison (black line). (**B**) Fractions spanning the elution volume 8-14 mL were analysed by SDS-PAGE and silver stained. Apo-*Sv*WhiD (48 µM) and $\sigma^{HrdB}{}_4$ (240 µM) were in 50 mM Tris, 300 mM NaCl pH 7.2. Note that the decrease in absorbance at 280 nm for *Sv*WhiD is due to the loss of the [4Fe-4S] cluster, which contributes significantly to $A_{280\ nm}$.

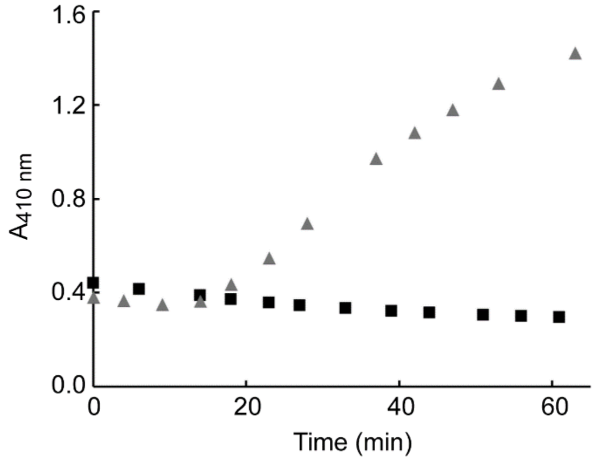

Figure S10. Sensitivity of *Sv*WhiD to O_2 is modulated by complex formation with σ^{HrdB}_4. Plot of $A_{410\ nm}$ as a function of time following exposure of *Sv*WhiD to O_2 in the absence (grey triangles) and presence (black squares) of σ^{HrdB}_4 (two-fold excess). *Sv*WhiD (25 µM) was in 50 mM Tris, 300 mM NaCl, pH 7.2 buffer.

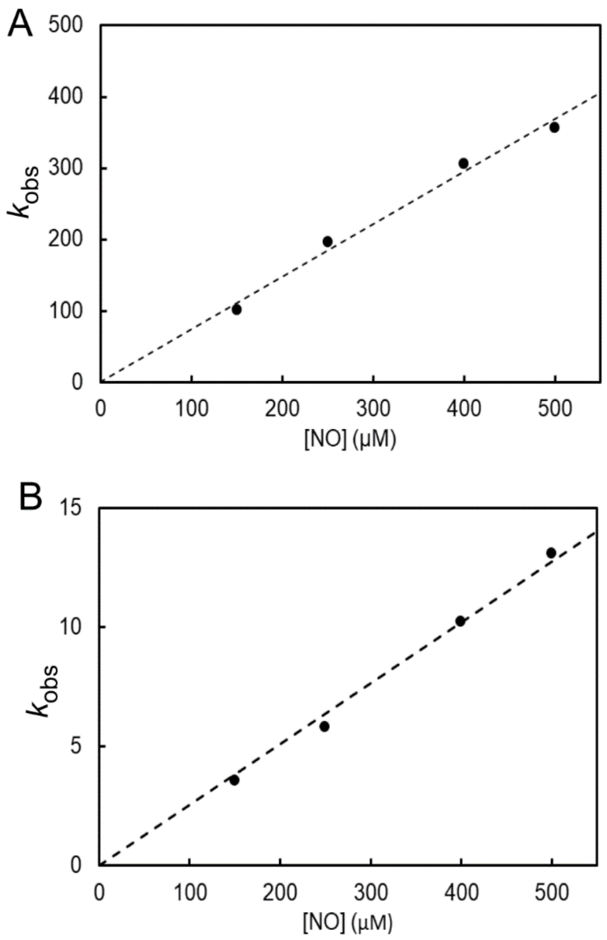

Figure. S11. Stopped-flow kinetic studies of the nitrosylation of the [4Fe-4S] cluster of *Sv*WhiD. Plots of observed rate constants for the first (**A**) and second **(B)** phases of $\Delta A_{360\ nm}$ following addition of varying concentrations of NO to *Sv*WhiD (Fig. 10 of the main paper). For both, a first order dependence was observed, indicating that the rate-limiting step of these reactions involves NO. Data are similar to those previously reported for nitrosylation reactions of other Wbl proteins (see main text). Experiments were performed with *Sv*WhiD (10 µM in [4Fe-4S]) in 50 mM Tris, 300 mM NaCl pH 7.2 at 25 °C.

Supporting references

30. Karimova, G., Pidoux, J., Ullmann, A., and Ladant, D. (1998) A bacterial two-hybrid system based on a reconstituted signal transduction pathway. *Proc. Natl. Acad. Sci. U.S.A.* **95**, 5752-5756.
